# Real-Time Assay as A Tool for Detecting *lytA* Gene in Streptococcus pneumoniae Isolates

**Published:** 2014-05-25

**Authors:** Masoud Hajia, Mohammad Farzanehkhah, Bahareh Hajihashemi, AliReza Dolatyar, Mohsen Imani, Roghieh Saburian, Marjan Rahnamaye Farzami, Mohammad Rahbar

**Affiliations:** 1 Reference Health Laboratories Research Center, Ministry of Health & Medical Education, Tehran, Iran; 2 Science and Research Branch, Islamic Azad University, Arak, Iran

**Keywords:** Streptococcus pneumoniae, lytA Gene, Real Time PCR

## Abstract

**Objective:**

In-time diagnosis of *Streptococcus pneumoniae (S. pneumonia)* can play a significant role in decreasing morbidity and mortality rate. Applying molecular methods has gained popularity due to the existing limits of routine diagnostic methods. Examining the expression of different genes of this bacterium through different molecular methods suggests that *lytA* gene has a higher sensitivity and specificity in diagnosis of *Streptococcus pneumoniae*. The aim of this study was to evalutate *lytA* gene expression in diagnosis of invasive *S. pneumonia* in culture positive specimens by real-time polymerase chain reaction (PCR).

**Materials and Methods:**

IIn this a descriptive study, All received specimens were isolated to identify *S. pneumoniae*. DNA was then extracted and after optimizing the test and determining the detection limit, samples were tested by real-time PCR using *lytA* gene primers.

**Results:**

Twenty seven isolates were diagnosed as *S. pneumoniae*. In all, the extracted DNA was positive in real-time method. The electrophoresis of the products also confirmed the presence of single product b along with the 53 base pair fragment. The detection limit of the test was less 6 colony forming unit (CFU).

**Conclusion:**

Real-Time PCR seems to provide reliable and rapid results. We suggest that this test should be conducted on the preliminary isolated specimens, since applying various biochemical tests need one extra working day.

## Introduction

The World Health Organization reports pneumococcal infections as the reason of 1.6 million deaths a year that over of one million of these are children under five (1, 2). For the ten countries with the greatest number of pneumococcal meningitis cases, the greatest estimate variability ranges from the jackknife analysis in Mexico and Brazil (reduction of 61 and 39%, respectively) to India, Nigeria, Pakistan, Bangladesh, Ethiopia, and Congo, where the biggest changes in case estimates were increased by 17-20% (3, 4).

In some neighboring countries meningitis death toll due to *Streptococcus pneumoniae* compared to other meningitis-causing bacteria has been reported as following: United Arab Emirates at 15.6%, Kuwait at 44%, Saudi Arabia at 35%, Iraq at 18.8% and Turkey at 29.7% (5-9).

Besides routine isolation in culture media, there are many different methods for detecting *S. pneumoniae* including latex agglutination, counter immunoelectrophoresis, and immunochromatography. The latex agglutination method which is used widely in comparison with other rapid methods has acceptable results (86% sensitivity) with fairly high sensitivity for Cerebral Spinal Fluid (CSF). Recently, various polymerase chain reaction (PCR) -based techniques are used to diagnose pneumococcus, the most important of which is real-time PCR (10- 12).

Three most applied pneumococcal genes are *lytA*, *ply*, and *psaA* that encode autolysin, Pneumolysin and surface adhesion A respectively, among all pneumococcal genes used in PCR (13, 14). The specificity levels for *lytA*, *psaA* and *ply* have been evaluated and reported at 100, 98 and 81% respectively (10). Some other genes such as *pbp1a* and *pbp2x* present in β-lactam -resistant Streptococcus and *cpbA* genes and wzy responsible for capsular genes can be also named (8, 13, 15). The box and *cpsA* genes are used as housekeeping genes.

The aim of this study was to set up a PCR protocol and to evaluate the *lytA* gene in those isolated invasive *S. pneumoniae* by the real-time PCR method on the collected specimens from Tehran and Isfahan provinces.

## Materials and Methods

### The study population

This was a descriptive study performed on isolated *S. pneumonia* from suspected patients. Overall, 27 samples were collected from pneumonia suspects and pneumococcus meningitis patients at Milad, Bahrami and Shariati Hospitals in Tehran and Alzahra, Amin and Mahdyieh Hospitals in Isfahan.

### Specimens and isolation

Received specimens were CSF, had eye discharge, pleural fluid and trachea aspirates. The mentioned samples were cultured on blood agar. Suspected colonies were identified by hemolysis pattern, sensitivity to optochin and bile solubility. All isolates were cultured in trypticase soy broth (TSB) media to preserve them which contained 10% glycerol and were maintained in the temperature of -70˚C (16).

### DNA erxtraction

DNA was extracted using the column based method from all the microbial suspensions by the DNA extraction kit (MiniPrep, QIAGEN GmbH, Germany).

### The quality control of the extracted samples

The optical density of all the extracted specimens were measured at the wavelengths of 260 and 280 nm and the ratio of 260 to 280 was determined for all extracted genomes.

### Real-time PCR

Primer pair and the probe were used from that reported by Carvalho et al. (10). Applied concentration of the PCR materials and amplification program were optimized (Tables1, 2). PCR was run by two real-time machines: Rotor gene 6000 (Corbett Research, Australia) and StepOne (Applied Biosystems, USA).

**Table 1:** Temperature program for real-time PCR


PCR Step	Temperature	Time	Cycle No.

**Initial Denaturing**	95°C	10 minutes	1 X
**Denaturing**	95°C	15 seconds	
**Annealing + Extension**	60°C	60 seconds	40 X


**Table 2:** Real-time PCR mix content


	Concentration	Volume

**Forward primer**	10 pmol	0.5 µl
**Reverse primer**	10 pmol	0.5 µl
**Probe**	10 pmol	0.5 µl
**MgCl_2_**	25 mM	1.5 µl
**dNTP Mix**	10 mM	0.5 µl
**10x Buffer**	10x	2.5 µl
**Taq polymerase**	5 U/µl	0.2 µl
**DNA**	10 µg/ml	2.0 µl
**D.D.W**	60˚C	17.0 µl


### Assessment of the detection limit

Serial 10-fold dilutions of pneumococcal reference strain ATCC 33400 serotype 5 were prepared. A serial 10 fold dilution was prepared and three drop of each dilution was put on blood agar medium. The lowest dilution that had the countable growing colony was detected and the number of the organisms was estimated in the starting ones. Each dilution was extracted and used in PCR as well.

### Electrophoresis

To ensure result accuracy, the PCR products were analyzed on 1% agarose gel with 100 base pair ladder. The specific band was observed after electrophoresis and staining with ethidium bromide.

## Results

### Detection limit of the test

**Fig 1:**
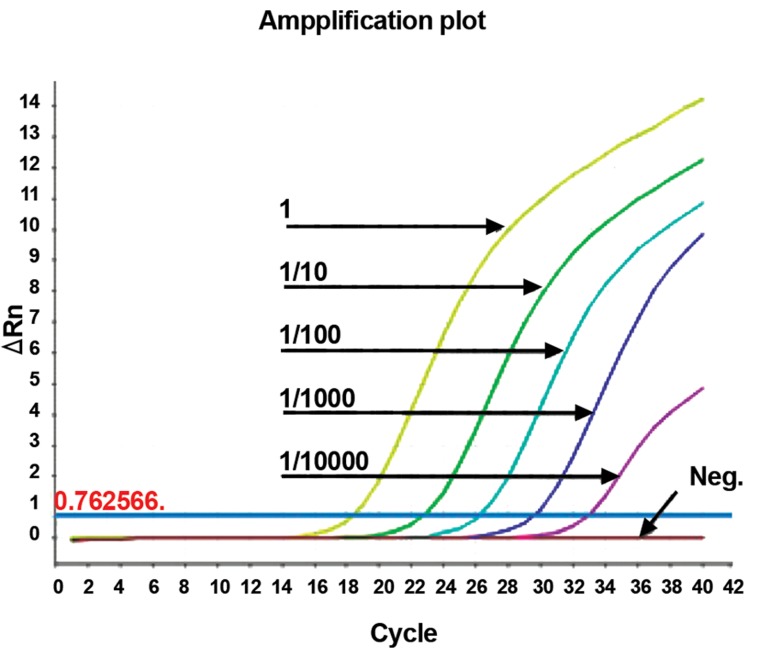
Serial dilution of positive sample’s graphs. The dilution 1, 1/10, 1/100, 1/1000, and 1/10000 are represent for 5.6×10^5^, 5.6×10^4^, 5.6×10^3^, 5.6×10^2^ and 5.6 CFU respectively.

The number of *S. pneumoniae* was estimated to be 5.6×10^6^ CFU in starting sample dilution. Real-time PCR was performed in each prepared tenfold dilution. Each dilution was extracted separately and was finally eluted in 50 μl of which 5 μl was used for the test. The extracted genome was tested by the real-time PCR protocols. The final sensitivity was detected to be 5.6 CFU (Fig 1).

### Specificity

Some non-pneumococcal bacteria causing respiratory tract infection (*H. influenzae* and *N. meningitides*) were used for the specificity test of the protocols as well as the noninvasive *S. pneumoniae*. The result of PCR was negative for all tested isolates. Besides, the BLAST search (NCBI) also revealed 100% specificity for a wide range of sequenced pneumococcal genomes.

### Sensitivity

**Fig 2:**
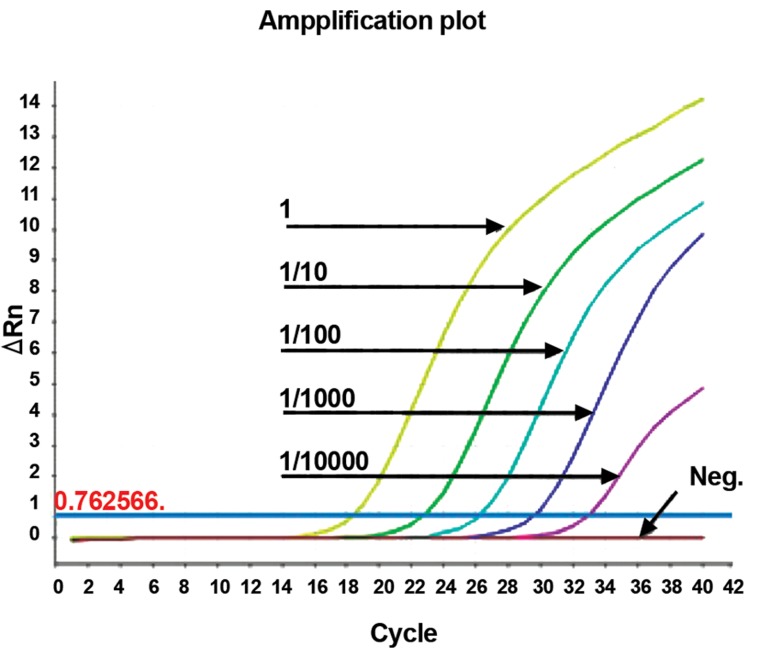
Real-time PCR graphs for samples.

**Fig 3:**
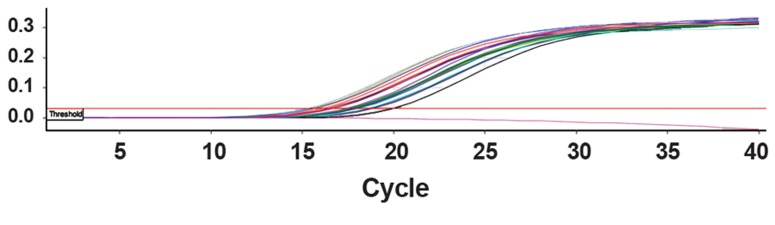
Electrophoresis of the PCR product.

All the collected specimens were identified as *S. pneumoniae* after rechecking by biochemical tests. All extracted specimens had an acceptable purity, since the ratio of measured optical density at 260 nm to 280 nm were between 1.8 and 2. All these isolates had positive signal in real-time PCR indicating 100 percent sensitivity. Their Ct was in the range of 16.1 and 20.3 (Fig 2). Also, a product band was observed in all tested specimens after electrophoresis (Fig 3).

## Discussion

Different *S. pneumoniae* genes have been used in different studies to detect this pathogen. The study where different genes (*erm, mef, pbp2b* and *pbp1a*) were analyzed and compared with each other was conducted by Nagai et al. (15). The PCR protocol proved presence of *lytA* gene in all tested specimens (16). In another study by Messmer et al. (17), *psaA* and *lytA* genes and two different sequence fragments of ply gene were analyzed and compared. The results of the ply gene were not satisfactory. From the 16 atypical streptococci samples, 8 from the first primer set and all the 16 from the second primer set were reported positive. The *psaA* gene showed difference only in one case but the *lytA* gene was detected in all the isolates.

Carvalho et al. (10) applied specific primers for *lytA*, *ply* and *psaA* genes. The results were compared for 67 *S. pneumoniae* isolated from 44 different serotypes and 3 non-capsule samples together with 104 non-pneumococcus isolates. All the 67 *S. pneumoniae* samples were positive for all the three genes which prove their appropriate sensitivity, although ply gene was positive in all non-capsule samples proving its lower specificity.

Analysis of results revealed specimens were positive in all isolates form both Tehran and Isfahan by this PCR protocol indicating 100% sensitivity. We used extracted genome of isolated organisms. It will be more important to apply the PCR directly on extracted genome of unknown specimens rather than isolated organisms, since it is required to access the results in the shortest possible time in some patients with critical conditions. We have to isolate and identify the organisms in received specimens in this study. We suggest to set up another project with its own fund to achieve directly tested specimens. Therefore, it is necessary to consider a second protocol as an internal control to ensure identification of true negative results in some specimens and distinguish them from false negative results.

It is shown that all pneumococcal specific type strains have 100 percent specificity based on BLAST search and other specificity tests. All extracted genomes had also similar positive signal when tested with real-time PCR. Although all our tested strains in this study have been isolated from various hospitals in Tehran and Isfahan, it is necessary to evaluate the sensitivity of this protocol with all known serotypes.

Real-time PCR has also being compared with the multiplex PCR format. Azzari et al. (12) applied 67 specimens for this comparison and reported higher sensitivity for realtime PCR (12).

## Conclusion

The molecular examination of *lytA* gene, due to its high sensitivity and specificity, is the best and most practical method to correctly diagnose invasive *S. pneumoniae* from clinical samples and isolates. However, the results must be confirmed in directly test specimens as well.

## References

[1] Werno AM, Murdoch DR (2008). Laboratory diagnosis of invasive pneumococcal disease. Clin Infect Dis.

[2] Harrison O, Brueggemann A, Caugant D, Ende A, Frosch M, Gray S (2011). Molecular typing methods for outbreak detection and surveillance of invasive disease caused by Neisseria meningitidis, Haemophilus influenzae and Streptococcus pneumonia, a review. Microbiology.

[3] O’Brien KL, Wolfson LJ, Watt JP, Henkle E, Deloria-Knoll M, McCall N (2009). Burden of disease caused by Streptococcus pneumoniae in children younger than 5 years: global estimates. Lancet.

[4] Mook-Kanamori BB, Geldhoff M, van der Poll T, van de Beek D (2011). Pathogenesis and pathophysiology of pneumococcal meningitis. Clin Microbiol Rev.

[5] Mahmoud R, Mahmoud M, Badrinath P, Sheek- Hussein M, Alwash R, Nicol AG (2002). Pattern of meningitis in Al-Ain medical district, United Arab Emirates--a decadal experience (1990-99). J Infect.

[6] Shabani IS, Al-Ateeqi W, Abu-Shanab O, El-Sori H, Omar N, Ahmed HF (2006). Childhood meningitis in Kuwait: epidemiology of etiologic agents and the need for pneumococcal disease prevention. Med Princ Pract.

[7] Al-Tawfiq JA, Abukhamsin A (2009). Burden and etiology of community-acquired bacterial meningitis in a hospital in Eastern Saudi Arabia: 1993-2005. Med Sci Monit.

[8] Al-Banae HZ, Habib KhA, Al-Khurki KA (2012). Occurrence of pneumococcal meningitis in Iraq. J Baghdad for Sci.

[9] Arda B, Sipahi OR, Atalay S, Ulusoy S (2008). Pooled analysis of 2,408 cases of acute adult purulent meningitis from Turkey. Med Princ Pract.

[10] Carvalho Mda G, Tondella ML, McCaustland K, Weidlich L, McGee L, Mayer LW (2007). Evaluation and improvement of real-time PCR assays targeting lytA, ply, and psaA genes for detection of pneumococcal DNA. J Clin Microbiol.

[11] Harris KA, Turner P, Green EA, Hartley JC (2008). Duplex real-time PCR assay for detection of Streptococcus pneumoniae in clinical samples and determination of penicillin susceptibility. J Clin Microbiol.

[12] Azzari C, Moriondo M, Indolfi G, Cortimiglia M, Canessa C, Becciolini L (2010). Realtime PCR is more sensitive than multiplex PCR for diagnosis and serotyping in children with culture negative pneumococcal invasive disease. PLoS One.

[13] Nomanpour B, Ghodousi A, Babaei T, Mousavi SA, Asadi S, Feizabadi M (2011). Detection and quantification of Streptococcus pneumoniae from Iranian patients with pneumonia and individual carriers by real time PCR. Afr J Biotechnol.

[14] Elberse KE, Nunes S, Sa -Leao R, van der Heide HG, Schouls LM (2011). Multiple-locus variable number tandem repeat analysis for Streptococcus pneumoniae: comparison with PFGE and MLST. PLoS One.

[15] Nagai K, Shibasaki Y, Hasegawa K, Davies TA, Jacobs MR, Ubukata K (2001). Evaluation of PCR primers to screen for Streptococcus pneumoniae isolates and beta-lactam resistance, and to detect common macrolide resistance determinants. J Antimicrob Chemother.

[16] Tedeschi R, De Paoli P (2011). Collection and preservation of frozen microorganisms. Methods Mol Biol.

[17] Messmer TO, Sampson JS, Stinson A, Wong B, Carlone GM, Facklam RR (2004). Comparison of four polymerase chain reaction assays for specificity in the identification of Streptococcus pneumoniae. Diagn Micr Infect Dis.

